# Ultralight Functionally Graded Hybrid Nanocomposites Based on Yttrium and Silica-Reinforced Mg10Li5Al Alloy: Thermal and Tribomechanical Properties

**DOI:** 10.3390/ma15249052

**Published:** 2022-12-18

**Authors:** Essam B. Moustafa, Emad Ghandourah, Rasha A. Youness, Ammar A. Melaibari, Mohammed A. Taha

**Affiliations:** 1 Mechanical Engineering Department, Faculty of Engineering, King Abdulaziz University, Jeddah 21589, Saudi Arabia; 2 Department of Nuclear Engineering, Faculty of Engineering, King Abdulaziz University, Jeddah 21589, Saudi Arabia; 3 Spectroscopy Department, National Research Centre, El Buhouth St., Dokki, Giza 12622, Egypt; 4 Solid State Physics Department, National Research Center, El Buhouth St., Dokki, Giza 12622, Egypt

**Keywords:** ultra-lightweight alloy, function graded composites, mechanical properties, wear rate, thermal properties

## Abstract

Despite the amazing properties of lightweight Mg_10_Li_5_Al alloy, its use in industrial applications is highly limited due to its low mechanical properties, wear resistance, and coefficient of thermal expansion (CTE). In this context, this work aimed to improve the above properties without sacrificing the important benefit of this alloy being lightweight. Therefore, function grade composites (FGCs) were prepared based on the Mg_10_Li_5_Al alloy reinforced by yttrium (Y) and silica fume using the powder metallurgy technique. Then, the nanocomposite’s microstructure, mechanical properties, artificial aging, wear resistance, and thermal expansion were examined. The results indicated that the precipitation (MgAlLi_2_), softening (AlLi_2_), and Mg_24_Y_5_ phases were formed in high-reinforced samples during high-energy milling. Furthermore, the addition of reinforcements accelerated the decomposition from the MgAlLi_2_ phase to the Al–Li phase (softening point). For the layer containing the highest reinforcement content, microhardness, strength, and Young’s modulus improved up to 40, 22.8, and 41%, respectively, due to the combined effect of the high strength of silica fume and the dispersion strengthening Mg_24_Y_5_ phase. Meanwhile, the same sample exhibited a remarkable improvement in wear rate and the CTE value to about 43 and 16.5%, respectively, compared to the non-reinforced alloy.

## 1. Introduction

Lightweight composites have become an important development direction for various fields, such as aviation, space transportation, and the automotive industries [1,2,3,4]. Magnesium–lithium (Mg-Li-X) alloys attract a great deal of interest in these uses because they are the lightest structural metals (densities of about 1.35–1.65 g/cm^3^) and have good strength besides their higher formability [5,6,7]. Despite these amazing features, there are several limitations to their actual applicability. One of the most serious issues is age softening, even at room temperature. For Mg-Li-X alloys, the aging behavior and phase transition process related to alloy composition and aging factors have yet to be determined [8]. Only Li can lower the density of alloys below that of Mg. It is also soluble up to 5.5 wt.% due to the formation of α- phase. The Li structure consists of a double structure, i.e., the α- and β-phases when the Li content is between 5.7 and 10.3 wt.%. In contrast, phase is present only if the Li concentration is greater than 10.3 wt.% [9].

In order to improve the strength of Mg–Li alloys without dropping the advantage of being lightweight, adding strengthening agents (such as rare earth elements and/or other alloying elements) or composite reinforcement is an excellent choice [9,10,11,12]. Based on this concept, Al has been preferred as the alloying element in the Mg–Li alloy thanks to its lighter weight and higher specific strength and corrosion resistance compared to the Mg–Li alloy. When a percentage of Al greater than 3% by weight is added, the strength of the alloy improves due to aging strengthening due to the formation of MgLi_2_Al and Al–Li intermetallic precipitation phases [9,13,14]. In Mg-Li-Al alloys, the MgLi_2_Al phase is generally considered a metastable strengthening phase, and the transition of metastable phases (from MgLi_2_Al to Al–Li) is believed to be a metastable strengthening phase that causes aging softening. The exact structure and evolution of the precipitate have not yet been determined. The intermetallic Mg_24_Y_5_ precipitate has recently been proven in the as-quenched state, although little attention has been paid to its evolution during aging. The detailed information on the strengthening precipitates in Mg-Li-Al alloys is currently of great interest [15,16]. Silica fume is a promising material for use as a reinforcement to increase the properties of metals and alloys mentioned earlier due to its high hardness, high strength, high modulus, and low cost [17,18].

Furthermore, due to grain refinement and the production of thermally stable rare earth-containing phases, Mg-Li-Al alloys modified by rare earth elements have lately attracted much attention [19,20]. Generally, to obtain gradient characteristics, function-graded composites (FGCs) are among the most attractive ways to achieve this goal. They have recently attracted a lot of attention and have seen a lot of progress in defense and aerospace applications. FGC is a type of composite created to meet certain industrial requirements [21]. FGCs are often compositional gradients of two materials or phases from one side of the sample to the other. This gradation allows for distinct gradient properties without a mechanically weak interface [22,23]. More importantly, there are several techniques for fabricating FGCs; powder metallurgy is one of the successful FGC preparation methods [24]. The powder metallurgy (PM) technique is a unique process in which a solid-state reaction occurs at room temperature between the fresh powder surfaces of reactant particles [25,26,27]. PM is a simple and effective method for achieving a uniform distribution of small inert particles in a fine-grained matrix. As a result, it may be utilized to make complex or impossible composites to prepare using traditional melting and casting methods. PM is usually conducted in three stages. The powders and balls are subjected to collisions with little breakage and plastic deformation. Finally, powders harden and fracture due to the constant increase in plastic deformation, resulting in new and smaller surfaces. There is a balance between cold welding and fracture in this step. As a result, it is possible to say that the “steady-state condition” has been achieved [28,29,30].

The mechanical properties and corrosion rates of various magnesium alloys have been improved by adding different types of rare earth elements such as gadolinium (Gd), erbium (Er), lanthanum (La), cerium (Ce), or neodymium (Nd) using the traditional casting process [31,32,33,34]. However, since the theoretical densities of these elements are high, i.e., 7.9, 9.2, 6.18, 6.76, and 7 g/cm^3^, one can expect that despite the improvement obtained in the mechanical and wear rates of Mg alloys, these alloys have lost their amazing benefit, i.e., their lightweight feature. Accordingly, this work aims to avoid this drawback by adding a low-density ceramic and a rare-earth element with different contents to prepare function-grade composites that preserve the important feature of Mg_10_Li_5_Al alloy and enhance its undesirable properties, such as low mechanical properties and poor wear rate. This addition will be followed by studies of the microstructure, particle size, aging behavior, and thermal expansion.

## 2. Experimental Setup

### 2.1. Preparation of Layer Powders of FGCs

To prepare the Mg_10_Li_5_Al alloy FGC-0, magnesium (Mg; 99.99%), lithium (Li; 99.95%), and aluminum (Al; 99.95%) powders with particle sizes of 80, 40, and 100 µm, respectively, were used as the raw materials of the matrix alloy. Then, yttrium (Y; 99.95%) and silica fume nanoparticles were used as reinforcements for Mg alloy with different weight percentages to prepare the different layers of FGCs. Noteworthy, the chemical composition of silica fume (wt.%) is listed in Table 1. FGCs consist of five composites (about 5 mm each) with different proportions of hybrid reinforcement and thickness. The batch compositions designed for the various layers of FGCs and their abbreviations are tabulated in Table 2. The various powder layers were subjected to high-energy milling for 10 h in an argon atmosphere with a rotation speed of 500 rpm and a ball-to-powder ratio (BPR) of 20:1, considering that the milling process took place in a cycle of 4 h and paused for 2 h. Stearic acid (0.1%) was also employed as a process control agent to prevent powder agglomeration during milling.

### 2.2. Characterization of Layer Powders of FGCs

X-ray diffraction (XRD; Philips PW) investigated the phase composition of the different layers. The micro-strain (ε) was determined from the full width at half maximum (B) of the peaks using Equation (1) as follows [24]:

(1) $$\varepsilon =\frac{B}{4tan\theta }$$

where θ is the angle in radians.

The morphology and particle sizes of the reinforcement and milled powders were characterized using high-resolution transmission electron microscopy (HRTEM; JEOL JEM-2100, Tokyo Big Sight, Japan).

### 2.3. Sinterability

**Figure 1:**
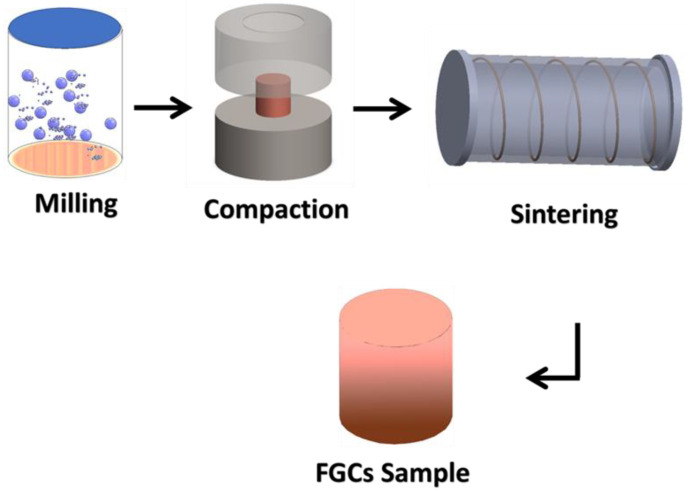
The schematic diagram represents the steps used for the preparation of FGCs.

**Figure 2:**
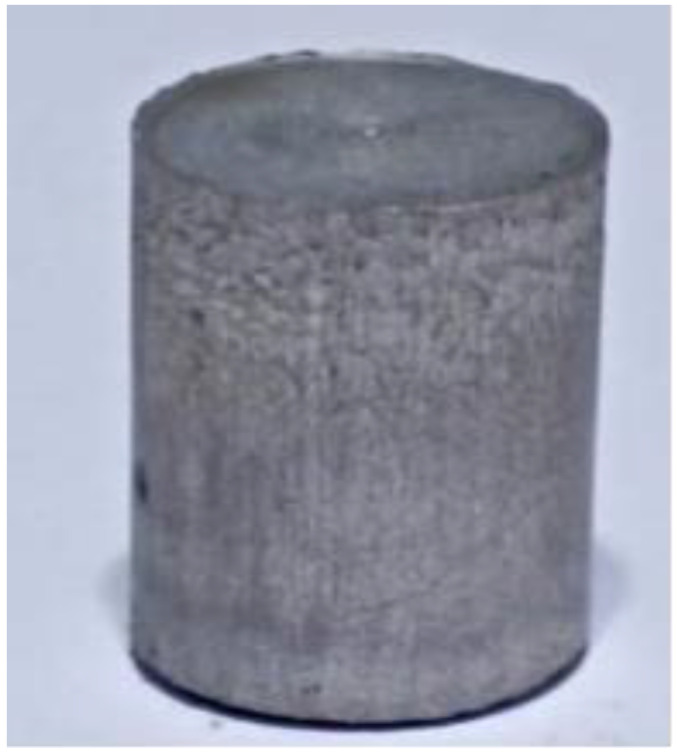
The typical image of the fabricated FGCs.

In order to sinter the prepared layers of FGC powders, they were first pressed by a hydraulic press using a load of 30 MPa, laying one layer next to the other, each layer having a thickness of 5 mm and a radius of 7.5 mm. After that, they were sintered at a temperature of 550 °C in argon gas for 2 h and a heating rate of 5 °C/min. The schematic diagram represents the steps for FGCs and the different layers prepared to obtain FGCs, as shown in Figure 1 and Figure 2, respectively.

### 2.4. Microstructure Analysis of Sintered Samples

Scanning electron microscopy (SEM; Quanta FEG25) was also carried out to examine the microstructure of the sintered samples. In addition, dispersive energy X-ray (EDX) was carried out to clarify the elemental analysis of the phases formed in the FGC-4 sample. 

### 2.5. Physical Properties

The bulk densities of the various FGCs were measured by the Archimedes method. Moreover, considering the theoretical densities for Mg, Li, Al, Y, and silica fume are 1.738, 0.534, 2.7, 4.472, and 1.45 g/cm^3^, respectively, the theoretical densities of the alloy and various layers were calculated by the mixture rule method. Hence, the relative densities of all layers were determined using bulk and theoretical densities.

### 2.6. Thermal Expansion

Thermal expansion of samples was measured from 30 to 500 °C using an automatic Netzsch DIL402 PC (Wittelsbacherstrasse, Germany) with a heating rate of 5 °C/min.

### 2.7. Mechanical Properties

As mentioned in our recent work [35,36], the microhardness of the sintered nanocomposites was measured by a Vickers tester according to ASTM B933-09 with an applied load of 1.9 N for 15 s. On the other hand, the sintered samples’ ultrasonic velocities, i.e., longitudinal (*V_L_*) and shear (*V_S_*), were measured using the pulse-echo technique. The values of Lame’s constants, i.e., λ and μ, were determined by [37,38]:

(2) $$\lambda =\rho \left(V_{L}^{2}-2V_{S}^{2}\right)$$

(3) $$\;µ\;=\rho V_{S}^{2}\;$$

where ρ is the bulk density of samples.

The values of the elastic modulus, longitudinal modulus (*L*), shear modulus (*G*), Young’s modulus (*E*), bulk modulus (*B*), and Poisson’s ratio (*ν*) were calculated using [39,40,41]:

(4) L=λ+2µ

(5) G=µ

(6) $$E=µ\frac{3\lambda +2µ}{\lambda +µ}$$

(7) $$B=\lambda +\frac{2}{3}µ$$

(8) $$\nu =\frac{\lambda }{2\left(\lambda +µ\right)}$$

The stress-strain compressive test of the layers has been examined according to ASTM E9–19. The ultimate strength and elongation are the maximum values of stress and strain on the curve, respectively. In order to study the precipitation hardening behavior, the layers were heated at 350 °C for 4 h and then quenched in water. Importantly, the temperature in the aging treatment was 100 °C and the aging time ranged from 0 to 150 h.

### 2.8. Wear Behavior

The various layers were subjected to a wear test under dry sliding conditions using a pin-on-disk wear-testing apparatus and specimens of 20 mm in diameter and 5 mm in height. The wear test process conditions included a speed of 0.8 m/s, a sliding distance of 300 m, and applied loads of 10 and 30 N. The wear rate (*W*) of sintered specimens was calculated by [42] the following equation:

(9) $$W\;\left(mm^{3}/km\right)=\frac{\;M\;}{\;\rho \times D}$$

where *M* represents the weight loss (g), *ρ* is the bulk density (g/mm^3^), and *D* (m) is the sliding distance (m).

## 3. Results and Discussion

### 3.1. Characterization of Layer Powders of FGCs

#### 3.1.1. TEM Observation

**Figure 3:**
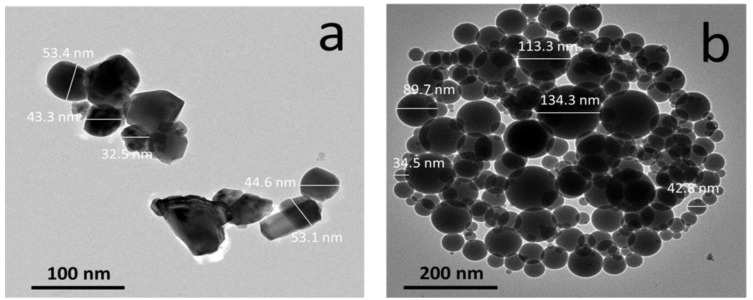
TEM images of (**a**) Y element and (**b**) Silica fume.

Figure 3 represents TEM images of the Y element and the ceramic reinforcement of the silica fume waste. The mean particle size of the Y element was about 45.4 nm, and the particles tended to be hexagonal with low agglomeration. In contrast, the silica fume particles had a spherical shape and their sizes ranged from 34.5 to 134.3 nm.

**Figure 4:**
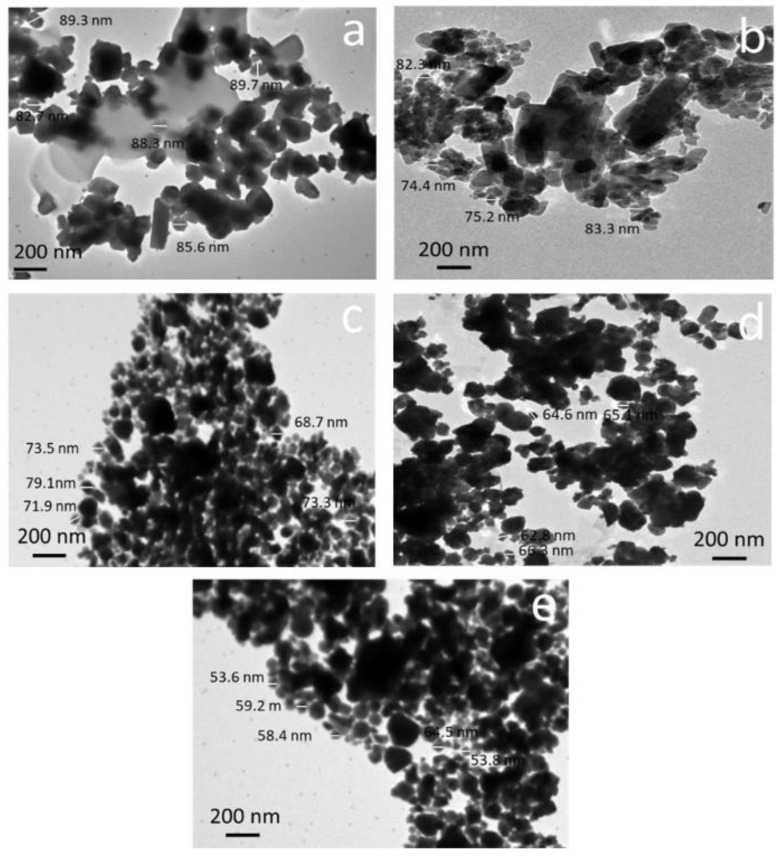
TEM images of (**a**) FGC-0, (**b**) FGC-1, (**c**) FGC-2, (**d**) FGC-3, and (**e**) FGC-4 powders after 20 h of milling.

**Figure 5:**
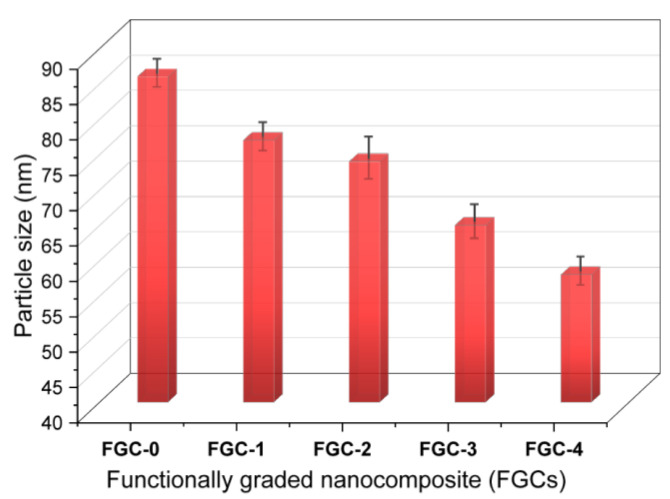
Particle sizes of FGC-0, FGC-1, FGC-2, FGC-3, and FGC-4 nanopowders after 20 h of grinding.

TEM images of layers FGC-0, FGC-1, FGC-2, FGC-3, and FGC-4 powders of FGCs after 20 h of milling are shown in Figure 4. As predicted, the FGC-0 (Mg-Li-Al alloy matrix) particles are deformed during milling. In contrast, the silica fume reinforcement particles are fragmented. Accordingly, for FGC-1, FGC-2, FGC-3, and FGC-4, the presence of silica fume is responsible for the observed reduction in particle sizes of the Mg alloy, as seen in the image. The presence of hard silica fume reinforcement lowers cold welding and speeds up the fracture process during milling, which explains the reduction in particle sizes. Furthermore, the reinforcement particles can insert between particles of the Mg alloy matrix, reducing their plastic deformability and making them more brittle, increasing the chances of fracture [41]. After milling, as seen in Figure 5, the main particle size value for FGC-0 is 85.6 nm. However, the main particle size values for FGC-1, FGC-2, FGC-3, and FGC-4 are 78.8, 73.1, 64.7, and 57.9 nm, respectively.

#### 3.1.2. XRD Analysis

**Figure 6:**
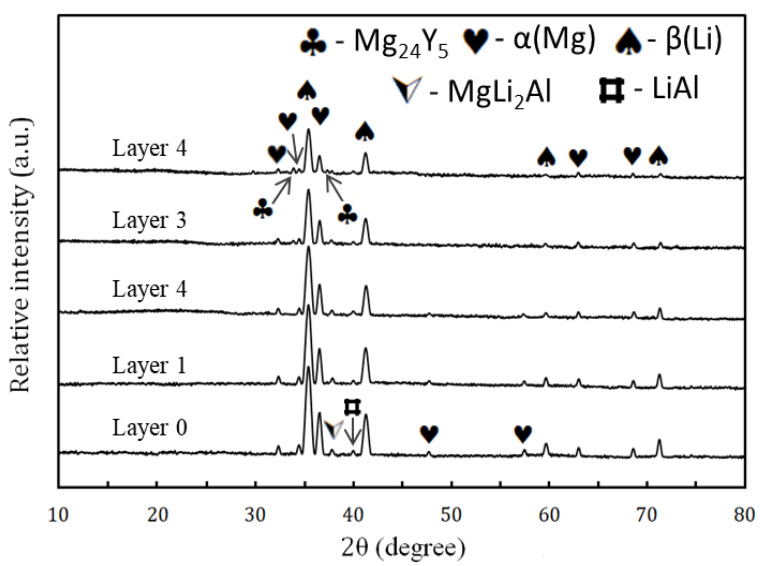
XRD patterns of different milled layers of FGCs.

**Figure 7:**
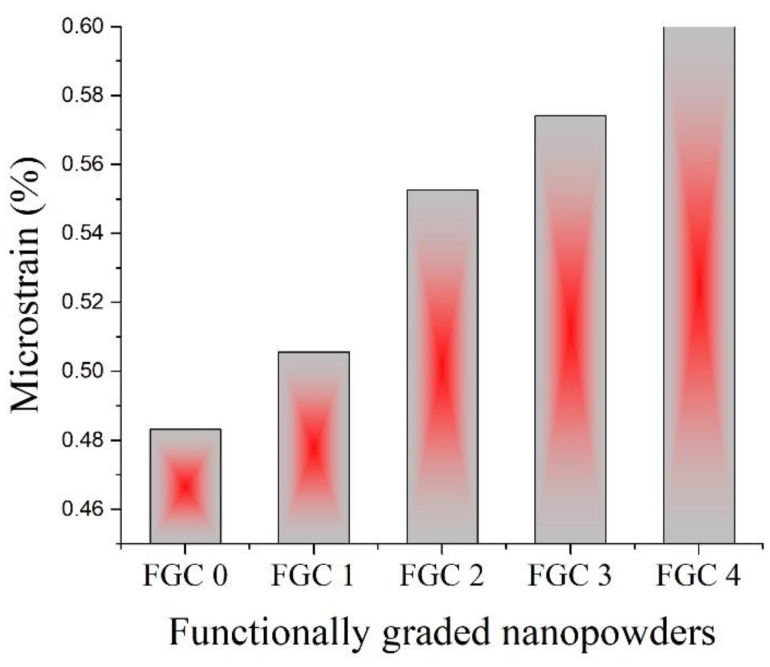
Micro-strain of different milled layers of FGCs.

Figure 6 shows the XRD patterns of the various milled layers of FGCs. It is evident from the XRD pattern of FGC-0 (alloy matrix) that it is mainly composed of a dual structure of α-Mg and β-Li phases. The α-Mg phase is present as identified from the XRD peaks at 2θ = 36.62°, 34.4°, 68.65°, 63.07°, 32.19°, and 47.82°, while the XRD peaks confirm the β-Li phase at 2θ = 35.39, 41.1, 59.53, and 71.2° according to the ICCD file cards 89-5003 and 89-4087, respectively. It is also clear that the intermetallic MgAlLi_2_ and Al–Li precipitated phases are confirmed by the presence of peaks located at 2θ = 38.51° and 44.76° according to the ICCD file cards 08-0283 and 71-0362, respectively. It should be noted that the gradually increasing weight percentage of Y, the Mg_24_Y_5_ intermetallic compound, is detected in FGC-3 and FGC-4 only. This observation can be expected from the general reader due to the lower weight percentage of Y in FGC-1 and FGC-2; therefore, the characteristic XRD peaks of this phase do not appear in the chart because they are below the detection limit of the XRD instrument. It can also be assumed that a steady increase in silica dust concentration leads to a noticeable decrease in the intensity and increases the full width at half the maximum of the peaks, causing the increase in micro-strain, as shown in Figure 7. They show an increase in width due to the hard reinforcing particles, which act as grinding balls and can cause a higher energy transfer to the Mg alloy matrix [28,43].

### 3.2. Microstructure Analysis of Sintered Samples by SEM

**Figure 8:**
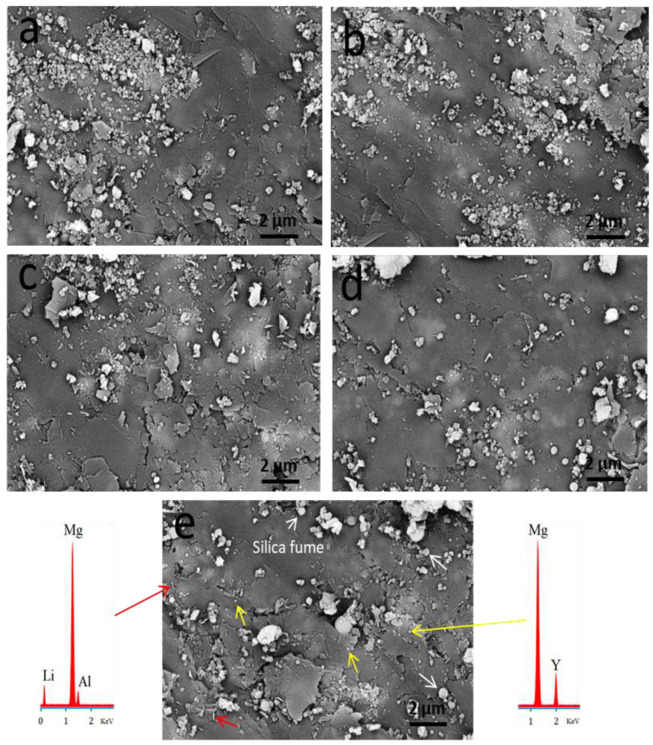
SEM images of sintered (**a**) FGC-0, (**b**) FGC-1, (**c**) FGC-2, (**d**) FGC-3, and (**e**) FGC-4 and their corresponding EDX pattern.

SEM images of all layers of FGCs are shown in Figure 8. It is evident from the figure that all layers exhibit excellent densification behavior, as indicated by the compact structure that appears and the almost complete absence of pores. This compaction can be attributed to the strong bonding between the Mg alloy particle matrix phases and considerable grain growth. Noteworthy, FGC-4 shows little porosity due to the higher weight percentage of the hybrid reinforcement, which helps to raise weak interfacial bonding between the Mg alloy and silica fume and reduce grain growth. Another important observation that can be seen in this figure is the homogeneous distribution of silica fume in addition to Mg_24_Y_5_ and MgLi_2_Al phases, which contribute to the improvement of the mechanical and wear rate of the FGCs layers, as will be discussed in the following sections. EDX was carried out to clarify the elemental analysis of the phases formed in the FGC-4 sample, namely Mg_24_Y_5_ and MgLi_2_Al. The silica foam is demonstrated as a white arrow in Figure 8e, while Li and Y elements appear as red and yellow color arrows, respectively.

### 3.3. Physical Properties

**Figure 9:**
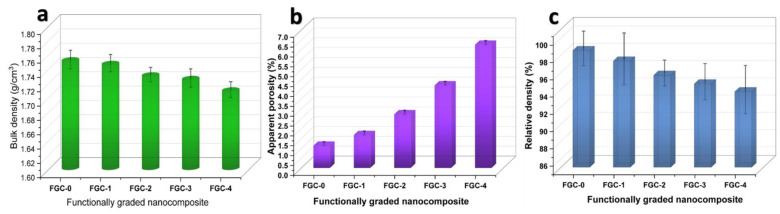
(**a**) Bulk density, (**b**) apparent porosity, and (**c**) relative density of FGCs sintered at 560 °C for 1 h in argon.

The density of sintered nanocomposites is well-known for its reliance on crystalline particle development and the removal of the majority of the sample’s porosity. At the same time, the presence of a secondary phase aids in the densification process and reduces residual porosity [44]. In this regard, the bulk density, relative density, and apparent porosity of FGCs layers sintered at 560 °C for 1 h in argon gas are measured and presented in Figure 9 a–c. It is clear that the apparent porosity of the FGCs showed a slight increase with the increase in the weight percentage of the hybrid reinforcement, while the bulk and relative density values decreased. It is known that the relative density was calculated using Equation (10):

ρ_relative_ = ρ_bulk_/ρ_theory_ (10)

The calculated theoretical densities of the FGCs, i.e., 0, 1, 2, 3, and 4, were 1.778, 1.794, 1.808, 1.822, and 1.823 g/cm^3^, respectively, calculated by the mixture rule. The density of the observed layers is very close to their theoretical density and contains few pores. Based on these results, the selected temperature is suitable for sintering these composites. This behavior is because, with the increase in the number of silica fume particles and the variation in phase composition (Mg_24_Y_5_, Al–Li, and MgAlLi_2_) in the layers of FGCs, the number of boundaries and barriers increase, which lead to less diffusion of the alloy particles and a decrease in the condensation behavior of the samples [45]. Similar results for the denser Mg alloys with enhanced density have been reported by other authors [46,47].

### 3.4. Thermal Expansion Behaviors

**Figure 10:**
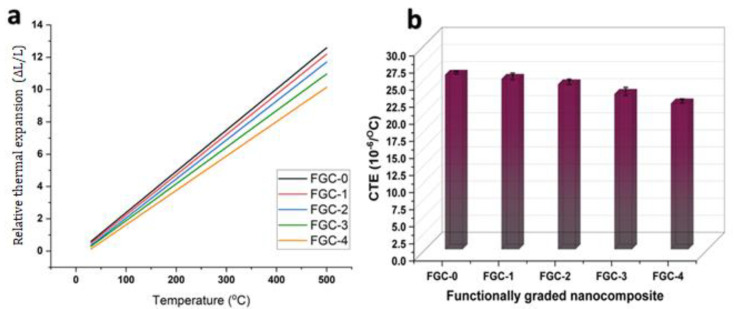
(**a**) Relative thermal expansion of FGC layers studied at 30−500 °C and (**b**) Variations in CTE value for different FGCs.

The relative thermal expansion (ΔL/L) of FGCs was studied at temperatures ranging from room temperature (i.e., 30 °C) to 500 °C, as represented in Figure 10. The results reveal that the value of ΔL/L for the layers with variable contents of Y and silica fume increased linearly with increasing temperature. On the contrary, the ΔL/L of the layers decreased with the increasing percentage weight of the hybrid reinforcements, especially at higher temperatures. For example, for FGC-0, 1, 2, 3, and FGC-4, the values of ΔL/L at 100 °C, are 2.38 × 10^−3^, 2.23 × 10^−3^, 2.05 × 10^−3^, 1.88 × 10^−3^, and 1.62 × 10^−3^, respectively. However, when the measurement temperature increases to 500 °C for the same FGC composite, the ΔL/L values are 12.59 × 10^−3^, 12.19 × 10^−3^, 11.70 × 10^−3^, 10.97 × 10^−3^, and 10.14 × 10^−3^, respectively. Figure 10 also shows the variation in the coefficient of thermal expansion (CTE) value of the different layers as it is considered the slope of the straight line of Figure 10.

The coefficient of linear thermal expansion (CTE) is a material parameter that indicates how much a substance expands when heated. The CTE values follow the same trend as thermal expansion, decreasing with increasing hybrid reinforcements, as shown in the graph. The obtained results indicate that the thermal expansion of FGCs is controlled by the competing interactions of the matrix expansion of the Mg alloy and the hybrid reinforcements and the constraints of the reinforcements at their interfaces. This observed decrease in the CTE value of the layers may be because the CTE of the matrix is greater than that of the hybrid reinforcement as the CTE value of the matrix components, including Mg, Li, and Al, is about 27 × 10–6, 22 × 10^−6^, and 23 × 10^−6^/°C, respectively, while for Y and silica fume, the value is about 10.6 × 10^−6^ and 0.9 × 10^−6^/°C, respectively. It is important to emphasize that the residual stresses caused by the thermal mismatch between the Mg alloy matrix and the hybrid reinforcements play a significant role in defining the thermal expansion behavior and increasing thermal stability in different layers of FGCs. These results agree with several works in the literature indicating a difference in CTE between metal matrix and reinforcement [36,48,49].

### 3.5. Mechanical Properties

**Figure 11:**
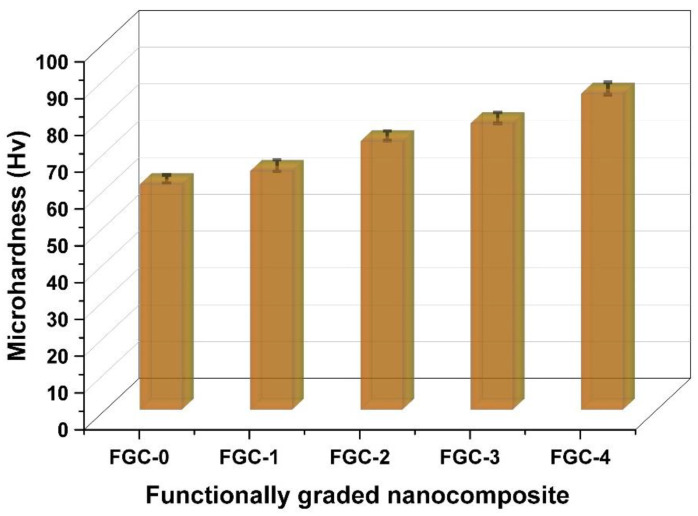
Microhardness of all FGCs sintered at 560 °C for 1 h in argon.

**Figure 12:**
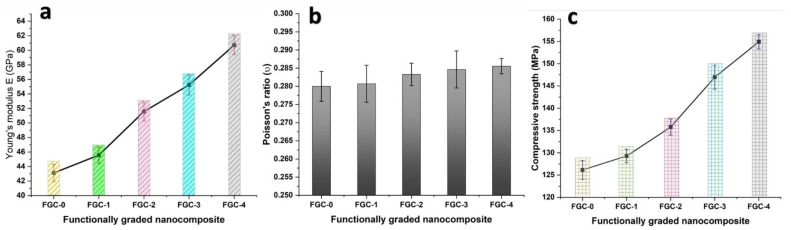
(**a**) Young’s modulus, (**b**) Poisson’s ratio as a representative of the elastic moduli, and (**c**) Compressive strength measured by non-destructive ultrasonic technique for all FGCs.

Microhardness is a particularly valuable feature since it provides information about the overall mechanical behavior of FGCs. Microhardness values can vary depending on many parameters, such as particle form, size, quantity, distribution, reinforcing density, and production procedure. Figure 11 shows the microhardness of sintered FGCs. As can be shown, increasing the hybrid reinforcement contents enhanced the microhardness values of the layers. The results for the longitudinal and shear velocities and the group of elastic moduli for various layers measured with the non-destructive ultrasonic technique are listed in Table 3. Additionally, for ease of comparison, Young’s modulus and Poisson’s ratio, as representatives of the whole group, are illustrated in Figure 12a. Remarkably, all the elastic moduli and ultrasonic velocities follow the same trend of microhardness. In other words, they increase as the contents of reinforcement increase. For example, the value of Young’s modulus for FGC-0 (i.e., the unreinforced layer) is 43.1 GPa, while for FGCs1, 2, 3, and 4, are 45.6, 51.6, 55.2 and 60.7 GPa, respectively.

The effect of weight percentages of reinforcement on the compressive strength of FGCs is represented in Figure 12c. Obviously, adding reinforcements to the matrix in the form of layers increases its compressive strength. The strength value of the layers was significantly increased from 126.16 to 154.91 MPa, (the percentage for such an increase is 23%) as a result of the incorporation of 4 wt.% of Y and 8 wt.% of silica fume (FGC-4). It is worth noting that despite the increase in porosity of the layers caused by increasing the reinforcements, the remarkable improvement in the mechanical properties of FGCs, such as microhardness, compressive strength, ultrasonic velocities, and elastic modulus, is attributed to the fact that the negative effect of porosity on mechanical properties is much less than the effect of adding hard reinforcement.

As a result of better dispersion of hard silica fume into layers of Mg alloy matrix, dislocation loops form around reinforcing particles, increasing the stress needed for further deformation [50,51]. Moreover, thermally induced residual stresses are caused by a substantial variation in the Mg alloy matrix and hybrid reinforcement CTE. The thermal stresses generated in the Mg alloy matrix contribute significantly to a high dislocation density in the interface region and thus strengthen the layers of the FGCs, even at small temperature variations (as shown in Figure 11) (thermal mismatch strengthening) [52]. Another important reason is that adding different weight percentages of the Y element to layers leads to forming the solid solution strengthening phase (Mg_24_Y_5_ phase) by effectively retarding the dislocation movement, which contributes to the strength enhancement. 

**Figure 13:**
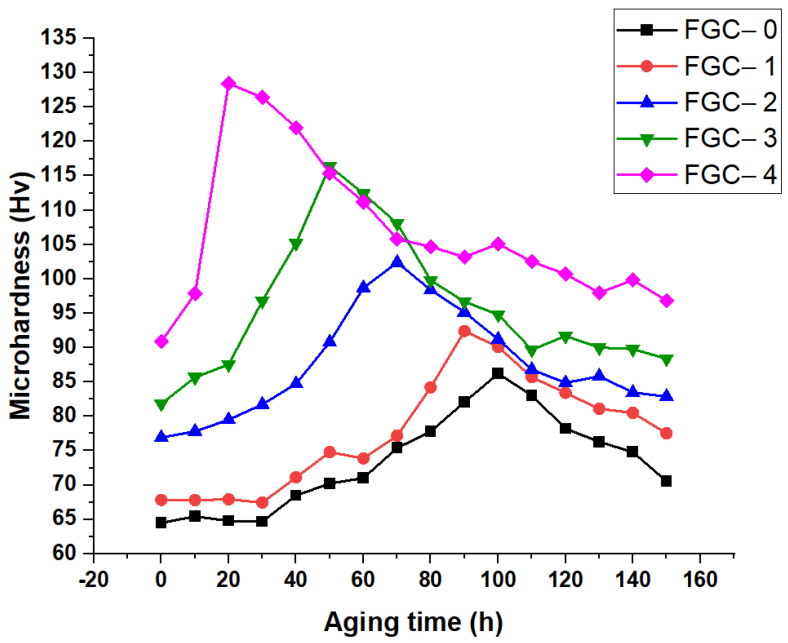
Effect of Y and silica fume on the aging-hardening behavior of FGCs aged at 180 °C.

The effect of Y and silica fume particles on the hardening behavior of FGCs aged at 180 °C is illustrated in Figure 13. Generally, the microhardness of various layers based on Mg alloy rises as the aging time goes on (hardening process). The aging curves then show a “softening point,” a turning point for alloy hardness (microhardness reduces with aging time after this point). It is known that the MgAlLi_2_ is a strengthening phase while the Al–Li is a softening phase. The precipitation of the MgAlLi_2_ and Mg_24_Y_5_ phases causes age hardening. The interaction between the precipitate and the dislocation, which generally includes the Orowan by passing and shearing mechanisms, causes precipitation intensification. Since MgAlLi_2_ is a metastable phase, it decomposes to the Al–Li phase during the further aging process. Furthermore, the time required to reach maximum microhardness, with the increase in microhardness value, decreases significantly with increasing hybrid reinforcement of the different layers. As the number of matrix-reinforcement interfaces and the amount of reinforcement increase, the aging kinetics of the coatings made of FGCs accelerate. For further clarification, one can note that in the case of FGC-1, age-hardening happens early in the aging process (within 5 h), followed by age-softening. As for FGC-1, 2, 3, and FGC-4, age-hardening occurs in 5, 4, 3, and 1 h, respectively, and then softens with aging. The results show that the hardness increases with increasing aging time until it reaches its maximum at 100 h and then decreases. This aging behavior is due to the formation of the Mg Li_2_Al precipitation phase during the alloy’s preparation.

### 3.6. Wear Behavior

**Figure 14:**
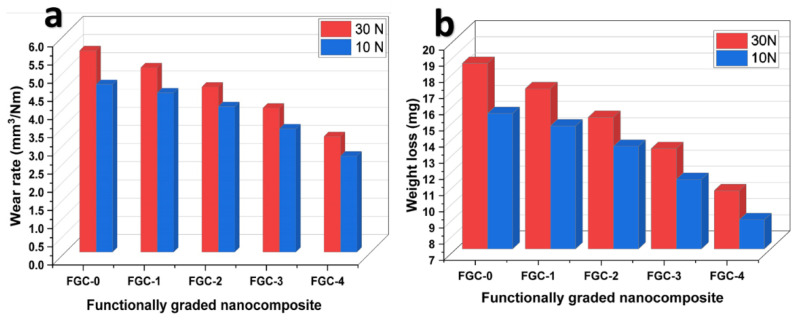
Effect of different hybrid reinforcements on the (**a**) wear rate of FGCs at 10 and 30 N and (**b**) weight loss.

Figure 14 illustrates the effect of different hybrid reinforcements on the weight loss and wear rate of FGCs layers at applied loads of 10 and 30 N. The results point out that the wear rate and weight loss of FGCs decreased with an increase in the hybrid reinforcement’s contents and increased with an increase in the applied load. It is interesting to see from the figure that an increase in the applied load during sliding wear tests is responsible for considerable increases in the weight loss and wear rate of all FGCs [53,54]. For example, the weight loss of FGCs from 0 to 4 when the applied load is 10 N is 15.37, 14.61, 13.34, 11.30, and 8.80 mg, respectively. The values of layer 1 wear rate at applied loads, i.e., 10 and 30 N, are 4.61 and 5.54 mm^3^/Nm, respectively, while the values of FGC-4 wear rate are 2.64 and 3.18 mm^3^/Nm, respectively. In other words, the latter layer’s weight loss and wear rate are reduced by about 11 and 33% compared to the non-reinforced layer Equation (11):

(11) $$Q=K\;\frac{W}{H}$$

where *Q* is the wear rate, *K* is a constant called wear coefficient, *W* is the applied load, and *H* is the microhardness of the sample. 

The lower value of the wear rate of layers with increased reinforcement can be attributed to the high microhardness compared to the non-reinforced coating and the good interfacial bonding between the hybrid reinforcement and the Mg alloy matrix. Another reason for the strong wear resistance of silica fume is that it can withstand the contact load between two surfaces, resulting in a soft Mg alloy matrix protection against wear. The wear rate of compacts is often substantially connected with the applied load, and as a result, it considerably influences wear behavior. According to this theory, increasing the applied load generates plastic deformation on the subsurface by increasing the counter-face penetration depth. Furthermore, when the applied load increases, the surface temperature rises, encouraging surface softening, resulting in more surface and subsurface damage, and resulting in greater weight loss.

## 4. Conclusions

- Using the powder metallurgy method to prepare function-graded composites (FGCs) based on Mg_10_Li_5_Al alloy and reinforced with different percentages of silica fume and yttrium (Y) element. The following are the main points to consider:
- The particle sizes of the layers were reduced with the addition of hybrid reinforcement. The addition of a rare earth element, i.e., Y, also led to the formation of the intermetallic compound, i.e., the Mg_24_Y_5_ phase, in addition to the presence of the MgAlLi_2_ and Al–Li phases bound to the Mg alloy;
- The homogenous distribution of silica fume and the existence of the Mg_24_Y_5_ phase in the various layers led to essential changes in the structure and properties of the FGCs, producing FGCs;
- The CTE value of the different layers of FGCs was greatly affected by the addition of silica fume and Y, which decreased with increasing reinforcement, reflecting their good thermal stability and ability to be better used in promising industrial applications;
- Except for elongation, adding hybrid reinforcements in different layers of FGCs significantly improved all mechanical properties. For instance, the recorded values of microhardness, compressive strength, and Young’s modulus increased to 39.7, 22.8, and 64%, respectively, for the layer with the highest percentage of high reinforcement. As expected, these results support using these FGCs in related applications;
- Compared with the non-reinforced layer, it is clear that the addition of hybrid reinforcements has accelerated the aging process of the reinforced layers;
- The weight loss and wear rate of the FGCs decreased with the content of hybrid reinforcement particles and increased with the applied load. The wear rate of FGC-4 under a 10 N applied load improved by about 42.7% compared to the original layer.

## Figures and Tables

**Table 1:** The chemical composition of silica fume (wt.%).

Element	SiO_2_	Al_2_O_3_	Fe_2_O_3_	CaO	MgO	SO_3_	K_2_O	Na_2_O	L.O.i
wt.%	92.9	0.67	2.40	0.91	1.05	0.86	0.17	0.94	0.5

**Table 2:** Batch design of the investigated FGCs (wt.%).

Nanocomposite	Mg_10_Li_5_Al Alloy	Y Element	Silica Fume
FGC-0	100	0	0
FGC-1	97	1	2
FGC-2	94	2	4
FGC-3	91	3	6
FGC-4	88	4	8

**Table 3:** Ultrasonic velocities and elastic moduli values of different layers of FGCs.

Nanocomposite	V_L_ (m/S)	V_S_ (m/s)	L (GPa)	E (GPa)	B (GPa)	G (GPa)	*ν*
FGC-0	5610.1	3101.2	55.1	43.1	38.3	16.8	0.2800
FGC-1	5779.3	3191.2	58.3	45.6	40.6	17.8	0.2807
FGC-2	6197.4	3407.8	66.4	51.6	46.4	20.1	0.2833
FGC-3	6428.2	3527.1	71.4	55.2	49.9	21.5	0.2846
FGC-4	6784.3	3717	78.7	60.7	55	23.6	0.2855
